# Poroelasticity derived from the microstructure for intrinsically incompressible constituents

**DOI:** 10.1007/s00033-025-02651-2

**Published:** 2025-12-19

**Authors:** R. Penta, C. Lonati, L. Miller, A. Marzocchi

**Affiliations:** 1 School of Mathematics and Statistics, University of Glasgow, Glasgow, UK; 2 Department of Mathematical Sciences “G.L. Lagrange”, Politecnico di Torino, Turin, Italy; 3 Department of Mathematics and Statistics, University of Strathclyde, Glasgow, UK; 4 Dipartimento di Matematica e Fisica “N. Tartaglia”, Università Cattolica del Sacro Cuore, Via della Garzetta 48, 25133 Brescia, Italy

**Keywords:** Poroelasticity, Homogenisation, Incompressibility, Saddle point problems, Partial differential equations, 74Q15, 35M10, 76S05

## Abstract

We provide a new derivation of the quasi-static equations of Biot’s poroelasticity from the microstructure via the asymptotic (periodic) homogenisation method (AHM) by assuming intrinsic incompressibility of both an isotropic, linear elastic solid and a low Reynolds’ number Newtonian fluid, in a small deformations regime. This is done by starting from a fluid–structure interaction (FSI) problem between the two phases at the pore scale, and by introducing both a solid and a fluid pressure, as both phases are equipped with an incompressibility constraint. Upscaling by the AHM then results in the expected Biot’s equation at the macroscopic scale, with coefficients which are to be computed by solving non-standard periodic cell problems at the pore scale. These latter differ from the ones arising from classical derivations of poroelasticity via the AHM, which are typically obtained by assuming that the elastic phase is compressible, and are characterised by a saddle point structure which is inherited from the equations governing the original FSI problem. The proposed approach, which is new and cannot be derived as a particular case of existing formulations, means that the poroelastic governing equations for intrinsic incompressible phases are obtained without performing any “a posteriori” assumption on the macroscale coefficients, as these latter are typically employed based on physical arguments rather than following from a rigorous analysis of the properties of the pore scale cell problems. The advantages of the current formulation for incompressible solids are as follows. (a) The formulation is derived for two genuinely incompressible phases and in particular for an incompressible solid, which means that a reduced number of input parameters is required to compute the effective stiffness. In the case of pore scale isotropy, this means that only the shear modulus is to be provided. (b) The pore scale cell problems can be solved without approximating the pore scale elastic properties to that of an incompressible solid, i.e. in the case of isotropy, no additional errors are to be introduced by utilising approximate values of the Poisson’s ratio (which in a compressible formulation can be close to, but not identical to, 0.5).

## Introduction

The most standard approach to determine the overall behaviour of deformable porous materials with fluid flowing in the pores relies on Biot’s theory of poroelasticity [7–10]. Biot’s governing equations were formulated by means of intuitive physical arguments, and therefore, the original formulation does not provide a link between the underlying structure of the pores and the macroscale response of the whole material.

The effective response of a deformable porous medium is described by (a) linearly relating the fluid velocity (relative to the solid one) averaged over the porous fluid domain with the macroscale pressure gradient, that is *Darcy’s law*, (b) linking macroscale pressure variations to solid and fluid volume changes consistently with the *mass balance of the medium*, and (c) featuring a constitutive equation that comprises an *additive decomposition of the stress* between a drained elastic response and the influence of the fluid pressure. In [1], the authors establish a link between the governing equations and the pore scale properties via the asymptotic homogenisation technique (see, e.g. [2–5, 29, 38, 49, 50, 52, 56] and references therein). This upscaling process translates a pore scale fluid–structure interaction problem into a macroscale problem governed by the equations of poroelasticity, while still retaining the microstructural complexity of the material. There are various other upscaling techniques such as mixture theory, effective medium theory and volume averaging. For an overview of these techniques, see for example [30] or [21].

Materials characterised by a poroelastic mechanical response exhibit an intrinsically multiscale structure. In particular, the average pore radius, which should be comparable to the distance between them for an approximately uniform pore distribution (*the pore scale*), is typically much smaller than the average size of the medium (*the macroscale*) which is effectively behaving as a poroelastic material. This scale separation allows us to apply the asymptotic homogenisation method.

There exists a large variety of scenarios of interest that can be treated by means of a poroelastic modelling approach, including soil mechanics [57], (bio) artificial constructs [13, 33, 34] and biological tissues, such as bone [18], organs, e.g. heart and lungs [6, 17, 37], as well as healthy and malignant cell aggregates [11].

The asymptotic homogenisation technique has been applied to derive Biot’s equations of poroelasticity in [12, 35, 52, 57]. The theory has been further developed in many ways, including growth of poroelastic materials [47], the addition of vascularised poroelastic materials [51], poroelastic composites [39], double poroelastic materials [40, 43], pre-stressed poroelastic materials [42] and solute transport [36]. Another development has consisted in considering poroelastic materials undergoing large deformations such as active poroelastic materials [15] and nonlinear poroelastic composites [41]. The technique has been also utilised to create poroelastic models of the myocardium incorporating electrical activity [44, 45].

In [12] the authors derive the standard Biot’s system of PDEs via asymptotic homogenisation and provide closed formulas for the coefficients of the model, as well as their associated pore scale differential problems. They extended the original formulation [1] to non-macroscopic uniformity and local boundedness, as this latter assumption is in general less strict than local periodicity.

While the classical derivation of poroelasticity from the microstructure, as in [12], can feature both the fluid and the solid intrinsic compressibility, the latter is normally not addressed via a mixed formulation, i.e. by considering the role of the solid pressure. In the former case, either the classical divergence-free constraint is enforced (for incompressible fluids), or time variations of the pressure are then related to the fluid bulk modulus (so that the divergence-free constraint is formally recovered for the fluid bulk modulus approaching infinity). In any case, the governing equations for the fluid phase explicitly include the role of the fluid pressure. On the other hand, the solid phase is normally dealt with by simply considering the balance equations for a linear elastic phase, and therefore, a mixed formulation, that is, featuring a constraint with its associated variable, is typically not considered. While this approach may seem *apparently* more general, the limit case of intrinsically incompressible phases, which implies the equivalence between the change of volume of the porous solid and the volume of fluid exchanged [24], cannot be rigorously obtained by imposing that the pore scale constituents are individually incompressible. Instead, the fact that variations of the solid and fluid volume balance each other for incompressible phases is obtained by considering the limit of relevant parameters, namely the Biot modulus and the Biot coefficient approaching infinity and one, respectively. However, the latter limits are performed on the basis of physical arguments rather than invoking the functional form of the parameters which arises from the asymptotic homogenisation upscaling and the corresponding cell problems. While it has been recently shown by the authors in [22] that the numerical solutions of the standard pore scale cell problems when approaching incompressibility of the individual phases leads to the expected results, this is reassuring, but does not constitute a rigorous proof of a limit behaviour based on the properties of the microstructure.

Therefore, in this work we focus on the derivation of the equations of poroelasticity for a material characterised by a low Reynolds’ number Newtonian incompressible fluid flowing in the pores of an incompressible solid matrix. We adopt a mixed formulation to describe both the fluid and the solid behaviour, such that the two sets of differential equations are both represented by saddle point Stokes’ problems [54] featuring their respective pressures. The problems mirror each other, as they both feature a divergence-free constraint in their respectively variables, i.e. the fluid velocity and the solid displacement, and heterogeneous material properties, namely the fluid viscosity and the shear modulus, respectively. We embrace the asymptotic homogenisation technique to derive the macroscale system of partial differential equations (PDEs) that describe the mechanical behaviour of a poroelastic material with intrinsically incompressible phases. The obtained result is new, and cannot formally be regarded as a particular case of standard formulations allowing for solid compressibility of the matrix such as [52]. The obtained balance equations are of Biot’s type, but feature pore scales problems which are different from the ones derived using standard approaches. This new formulation automatically addresses the properties of a macroscale system of PDEs representing the behaviour of a poroelastic material with intrinsically incompressible constituents, and thus represents the rigorous upscaled limit in this case.

The paper is organised as follows. We begin by introducing the fluid–structure interaction problem in Sect. 1. This problem describes the interactions between an incompressible elastic matrix and an incompressible fluid that is flowing in the pores. We non-dimensionalise the FSI problem in Sect. 2. We then introduce the *asymptotic homogenisation method* (AHM) in Sect. 3. In Sect. 4, we apply the AHM to the non-dimensional multiscale system of PDEs derived in Sect. 3 to obtain the macroscale PDEs governing the homogenised mechanical behaviour of a poroelastic material with intrinsically incompressible constituents. Finally in Sect. 5, we conclude by discussing the potential, limitations, and further perspectives of our work.

## The fluid–structure interaction problem

We start by identifying our physical domain with a set $\Omega \subset \mathbb {R}^3$ where $\Omega$ consists of the disjoint union of a porous solid matrix $\Omega _s$ and a connected fluid compartment $\Omega _f$ such that ${\bar{\Omega }}={\bar{\Omega }}_s \cup {\bar{\Omega }}_f$. We assume a structure where the typical length scale of the pores, denoted by *d*, is small compared to the size of the domain, which we denote by *L*. This means that their ratio $\epsilon$ satisfies

1 $$\begin{aligned} \frac{d}{L}=\epsilon \ll 1. \end{aligned}$$

The equilibrium equation for the solid matrix in absence of inertia and body forces reads:

2 $$\begin{aligned} \nabla \cdot {\textsf{T}}_s={\textbf{0}} \quad \text{ in } \quad \Omega _s, \end{aligned}$$

where ${\textsf{T}}_s$ is the solid matrix Cauchy stress tensor. We assume that the solid matrix is a linear elastic, isotropic, strictly incompressible solid, thus characterised by the following constitutive relationship:

3 $$\begin{aligned} {\textsf{T}}_s= -p_s {\textsf{I}}+2 \mu _s \xi {({\textbf{u}})}, \end{aligned}$$

where ${\textbf{u}}$ is the elastic displacement in the solid matrix, while $p_s$ and $\mu _s$ are the solid pressure and shear modulus, respectively. We remark that since we are addressing a small deformations regime, it is not necessary to distinguish between the current and reference configuration, see, e.g. [28] among many others. However, this is crucial when dealing with finite deformations, see, e.g. [41] for an example of poroelastic upscaling of a nonlinear fluid–structure interaction problem via asymptotic homogenisation. The symmetric gradient operator $\xi$ is defined as:

4 $$\begin{aligned} \xi ({\cdot })=\frac{\nabla {(\cdot )} +(\nabla {(\cdot )})^{\mathrm T}}{2}. \end{aligned}$$

The incompressibility constraint for the solid phase reads

5 $$\begin{aligned} \nabla \cdot \textbf{u}=0\quad \text{ in } \quad \Omega _s. \end{aligned}$$

### Remark 1

(Anisotropic materials) We note that in general constitutive relationship (3) is the special case of the more general anisotropic constitutive relationship of the form ${\textsf{T}}_s= -p_s {\textsf{I}}+\mathbb {C}: \xi {({\textbf{u}})}$, where $\mathbb {C}$ could in principle represent a fourth rank tensor equipped with standard major and minor symmetries, while being set to $2 \mu _s \mathbb {I}^{S}$ in (3), where $\mathbb {I}^{S}$ is the fourth rank *symmetric* identity tensor, that is the identity operator when applied to symmetric second rank tensors. The operation “ : ” is the standard double contraction between tensors of rank two or higher. In particular, for a generic vector, and hence in particular for ${\textbf{u}}$, the symmetric identity tensor satisfies $\mathbb {I}^{S}:\nabla {{\textbf{u}}}=\mathbb {I}^{S}:\xi ({\textbf{u}})=\xi ({\textbf{u}})$, and component-wise reads:

6 $$\begin{aligned} I_{ijkl}^{S}=\frac{1}{2}(\delta _{ik}\delta _{jl}+\delta _{il}\delta _{jk}). \end{aligned}$$

However, for the sake of a smoother presentation of the results, we adopt relationship (6) as it leads to a standard Stokes’ problem that formally mirrors the Stokes’ problem for incompressible fluids which is introduced right below. In addition, this simplified approach is embraced to focus on the actual recovery of incompressible poroelasticity rather than issues related to the properties and number of independent parameters arising from incompressible anisotropic elasticity, which will be the focus of future work. However, the structure of the illustrated steps is performed to pave the way for straightforward generalisation to more general anisotropic scenarios.

In the fluid compartment, we are considering a low Reynolds’ number Newtonian fluid, so that the balance of stresses can be written as follows:

7 $$\begin{aligned} \nabla \cdot {\textsf{T}}_f={\textbf{0}}\quad \text{ in } \quad \Omega _f, \end{aligned}$$

where ${\textsf{T}}_f$ is the fluid stress tensor defined by

8 $$\begin{aligned} {\textsf{T}}_f= -p_f {\textsf{I}}+2 \mu _f \xi {({\textbf{v}})} \end{aligned}$$

and ${{\textbf{v}}}$, $p_f$, and $\mu _f$ denote the fluid velocity, pressure and viscosity, respectively. The incompressibility constraint for the fluid phase reads

9 $$\begin{aligned} \nabla \cdot \textbf{v}=0\quad \text{ in } \quad \Omega _f. \end{aligned}$$

We now need to close the fluid–structure interaction problem between the solid matrix and the fluid by specifying appropriate boundary conditions. We first prescribe appropriate conditions across the interface between $\Omega _s$ and $\Omega _f$, which we define as

10 $$\begin{aligned} \Gamma :=\partial \Omega _s \cap \partial \Omega _f. \end{aligned}$$

Continuity of both velocities and tractions across the interface $\Gamma$ then yields

11 $$\begin{aligned} \dot{{{\textbf{u}}}}= {\textbf{v}}&\qquad \qquad \text{ on }\quad \Gamma \end{aligned}$$

12 $$\begin{aligned} {\textsf{T}}_s {\textbf{n}}={\textsf{T}}_f {\textbf{n}}&\qquad \qquad \text{ on }\quad \Gamma , \end{aligned}$$

where $\dot{\textbf{u}}$ is the solid velocity.

### Remark 2

(Macroscale boundary conditions) In order to close the problem (2, 5), (7, 9), as supplemented by the constitutive equations (3) and (8), we must also prescribe boundary conditions on the external boundary of the domain, i.e. on $\partial \Omega$, in addition to the interface conditions (11, 12). These conditions could be, for example, of Dirichlet–Neumann type, as noted in [55]. The conditions on the external boundary generally do not play any role in the derivation of results via application of formal asymptotic homogenisation, although they are important in the context of rigorous two-scale convergence, see, e.g. [14].

### Remark 3

(Heterogeneous properties of the material) We are herein assuming that each variable is in general varying both in space and time, that is ${\textbf{u}} ({\textbf{x, }}\textrm{t})$, ${\textbf{v}}({\textbf{x, }}\textrm{t})$, $p_s ({\textbf{x, }}\textrm{t})$, and $p_f ({\textbf{x, }}\textrm{t})$, and that the material properties are heterogeneous, i.e. $\mu _f ({\textbf{x}})$ and $\mu _s ({\textbf{x}})$. However, if we consider the particular case of constant shear modulus $\mu _s$ and fluid viscosity $\mu _f$, then the stress balance equations can also be formally rewritten as two standard Stokes’ problems for constant coefficients featuring the Laplacian of the elastic displacement ${\textbf{u}}$ and the fluid velocity ${\textbf{v}}$. This can be deduced by substituting the constitutive relationships (3) and (8) in the stress balance equations (2) and (7), respectively, and by taking into account both incompressibility constraints (5) and (9), obtaining

13 $$\begin{aligned} \mu _s \Delta \textbf{u}= \nabla p_s \quad \text{ in } \quad \Omega _s, \end{aligned}$$

and

14 $$\begin{aligned} \mu _f \Delta \textbf{v}= \nabla p_f \quad \text{ in } \quad \Omega _f. \end{aligned}$$

In the next section, we perform a non-dimensional analysis of Eqs. (2–5), (7–9), and (11–12).

## Non-dimensional analysis

We now perform a non-dimensional analysis of the system of PDEs at hand. In particular, we set

15 $$\begin{aligned} \begin{aligned}&\mathbf{{x}}=L\mathbf{{x}'}, \quad \nabla =\frac{1}{L}\nabla ',\quad {\textbf{u}}=L{\textbf{u}}', \\&{\textbf{v}}=\frac{Cd^2}{\mu _{ref}}{{\textbf{v}}'}, \quad p_s=CLp'_s, \quad p_f=CLp'_f, \end{aligned} \end{aligned}$$

where *C* is a reference pressure gradient and $\mu _{ref}$ a reference viscosity. Then using (15), Eqs. (2–5), (7–9) and (11, 12) can be rewritten in non-dimensional form as follows

16 $$\begin{aligned} \nabla ' \cdot {\textsf{T}}'_s={\textbf{0}}&\qquad \qquad \text{ in } \quad \Omega _s \end{aligned}$$

17 $$\begin{aligned} \nabla ' \cdot {\textbf{u}}'=0&\qquad \qquad \text{ in } \quad \Omega _s \end{aligned}$$

18 $$\begin{aligned} \nabla ' \cdot {\textsf{T}}'_f={\textbf{0}}&\qquad \qquad \text{ in } \quad \Omega _f\end{aligned}$$

19 $$\begin{aligned} \nabla ' \cdot {\textbf{v}}'=0&\qquad \qquad \text{ in } \quad \Omega _f\end{aligned}$$

20 $$\begin{aligned} \dot{{\textbf{u}}}'= {\textbf{v}}'&\qquad \qquad \text{ on }\quad \Gamma \end{aligned}$$

21 $$\begin{aligned} {\textsf{T}}'_s {\textbf{n}}={\textsf{T}}'_f {\textbf{n}}&\qquad \qquad \text{ on }\quad \Gamma , \end{aligned}$$

where the characteristic time is *L*/*V*, with the characteristic velocity $V = C d^{2} / \mu _{ref}$ ; the non-dimensional stress tensors are given by

22 $$\begin{aligned} {\textsf{T}}'_s=-p'_s {\textsf{I}}+2 \bar{\mu }_s \xi '{({\textbf{u}}')}&\qquad \qquad \text{ in }\quad \Omega _s\end{aligned}$$

23 $$\begin{aligned} {\textsf{T}}'_f= -p'_f {\textsf{I}}+2 \epsilon ^2 \bar{\mu }_f \xi '{({\textbf{v}}')}&\qquad \qquad \text{ in } \quad \Omega _f, \end{aligned}$$

where $\displaystyle \xi ' ({\cdot })$ is the non-dimensional symmetric gradient operator and the non-dimensional shear modulus and viscosity are given by

24 $$\begin{aligned} \bar{\mu }_s=\frac{\mu _s}{CL},\quad \bar{\mu }_f=\frac{\mu _f}{\mu _{ref}}, \end{aligned}$$

respectively. In the next section, we illustrate the asymptotic homogenisation method, see, e.g. [2, 4, 29, 38, 50] and its application to the system of non-dimensional PDEs (16–21) by also omitting the primes (for the remainder of this work) for the sake of simplicity of notation.

## The asymptotic homogenisation method

We commence by enforcing the pronounced length scale separation that exists in the system between the microscale and the macroscale, cf. (1), in order to introduce a new *microscale* variable in terms of the scale separation parameter $\epsilon$. That is,

25 $$\begin{aligned} \textbf{y}=\frac{\textbf{x}}{\epsilon }, \end{aligned}$$

which addresses local spatial variations, i.e. those which are taking place at the pore scale level.

### Spatial variations decoupling

From now on, we further assume that every material property depends on both the *macroscale* ${\textbf{x}}$ and the microscale ${\textbf{y}}$, i.e. $\bar{\mu }_s ({\textbf{x}}, {\textbf{y}})$, $\bar{\mu }_f ({\textbf{x}}, {\textbf{y}})$. We further assume that every variable exhibits both microscale and macroscale spatial variations of the fields. As such, the gradient operator $\nabla (\cdot )$, its symmetric part $\xi (\cdot )$ and the divergence $\nabla \cdot (\cdot )$ transform in terms of their microscale and macroscale counterparts according to (25) and the chain rule, i.e.

26 $$\begin{aligned} \nabla _{{\textbf{x}}} (\cdot )+\frac{1}{\epsilon }\nabla _{{\textbf{y}}} (\cdot ), \quad \xi _{{\textbf{x}}} (\cdot )+\frac{1}{\epsilon }\xi _{{\textbf{y}}} (\cdot ), \quad \nabla _{{\textbf{x}}} \cdot (\cdot )+\frac{1}{\epsilon }\nabla _{{\textbf{y}}} \cdot (\cdot ), \end{aligned}$$

respectively, where in particular

27 $$\begin{aligned} \xi _{{\textbf{x}}} ({\cdot })=\frac{\nabla _{{\textbf{x}}}{(\cdot )} +(\nabla _{{\textbf{x}}}{(\cdot )})^{\mathrm T}}{2}, \quad \xi _{{\textbf{y}}} ({\cdot })=\frac{\nabla _{{\textbf{y}}}{(\cdot )} +(\nabla _{{\textbf{y}}}{(\cdot )})^{\mathrm T}}{2}. \end{aligned}$$

### The multiscale system of PDEs

We now obtain a multiscale system of PDEs by considering (16–21) and applying the transformation of differential operators (26), which yields

28 $$\begin{aligned} \nabla _{{\textbf{y}}} \cdot {\textsf{T}}^{\epsilon }_s +\epsilon \nabla _{{\textbf{x}}} \cdot {\textsf{T}}^{\epsilon }_s={\textbf{0}}&\qquad \qquad \text{ in } \quad \Omega _s\end{aligned}$$

29 $$\begin{aligned} \nabla _{{\textbf{y}}} \cdot {\textbf{u}}^{\epsilon }+\epsilon \nabla _{{\textbf{x}}} \cdot {\textbf{u}}^{\epsilon }=0&\qquad \qquad \text{ in } \quad \Omega _s\end{aligned}$$

30 $$\begin{aligned} \nabla _{{\textbf{y}}} \cdot {\textsf{T}}^{\epsilon }_f +\epsilon \nabla _{{\textbf{x}}} \cdot {\textsf{T}}^{\epsilon }_f={\textbf{0}}&\qquad \qquad \text{ in } \quad \Omega _f\end{aligned}$$

31 $$\begin{aligned} \nabla _{{\textbf{y}}} \cdot {\textbf{v}}^{\epsilon }+\epsilon \nabla _{{\textbf{x}}} \cdot {\textbf{v}}^{\epsilon }=0&\qquad \qquad \text{ in } \quad \Omega _f\end{aligned}$$

32 $$\begin{aligned} \dot{{\textbf{u}}}^{\epsilon }= {\textbf{v}}^{\epsilon }&\qquad \qquad \text{ on }\quad \Gamma \end{aligned}$$

33 $$\begin{aligned} {\textsf{T}}^{\epsilon }_s {\textbf{n}}={\textsf{T}}^{\epsilon }_f {\textbf{n}}&\qquad \qquad \text{ on }\quad \Gamma , \end{aligned}$$

supplemented by the following multiscale constitutive relationships

34 $$\begin{aligned}&\phantom {a}\epsilon {\textsf{T}}^{\epsilon }_s=-\epsilon p^{\epsilon }_s {\textsf{I}}+2 \bar{\mu }_s \xi _{{\textbf{y}}}{({\textbf{u}}^{\epsilon })}+2 \epsilon \bar{\mu }_s \xi _{{\textbf{x}}}{({\textbf{u}}^{\epsilon })}\quad \text{ in }\quad \Omega _s\end{aligned}$$

35 $$\begin{aligned}&{\textsf{T}}^{\epsilon }_f= -p^{\epsilon }_f {\textsf{I}}+2 \epsilon \bar{\mu }_f \xi _{{\textbf{y}}}{({\textbf{v}}^{\epsilon })}+2 \epsilon ^2 \bar{\mu }_f \xi _{{\textbf{x}}}{({\textbf{v}}^{\epsilon })} \quad \text{ in } \quad \Omega _f. \end{aligned}$$

The notation with the superscript $\epsilon$ attached to the relevant fields $p_f$, $p_s$, ${\textbf{u}}$, ${\textbf{v}}$, ${\textsf{T}}_f$, and ${\textsf{T}}_s$ indicates that they are now depending on $\epsilon$, and, as it is usually assumed in the context of formal asymptotic homogenisation, we assume that this dependency can be represented as regular power series expansions, i.e.:

36 $$\begin{aligned} \begin{aligned}&p^{\epsilon }_{f, s}(\textbf{x,y},t)=\sum _{l=0}^\infty p_{f, s}^{(l)}(\textbf{x,y},t)\epsilon ^l, \\&{\textsf{T}}^{\epsilon }_{f, s}(\textbf{x,y},t)=\sum _{l=0}^\infty {\textsf{T}}_{f, s}^{(l)}(\textbf{x,y},t)\epsilon ^l, \\&{\textbf{u}}^{\epsilon }(\textbf{x,y},t)=\sum _{l=0}^\infty {\textbf{u}}^{(l)}(\textbf{x,y},t)\epsilon ^l, \\&{\textbf{v}}^{\epsilon }(\textbf{x,y},t)=\sum _{l=0}^\infty {\textbf{v}}^{(l)}(\textbf{x,y},t)\epsilon ^l. \end{aligned} \end{aligned}$$

### Geometrical assumptions

In this subsection, we outline simplifying geometrical assumptions concerning the geometry of the pore structure under consideration.

**Fig. 1:**
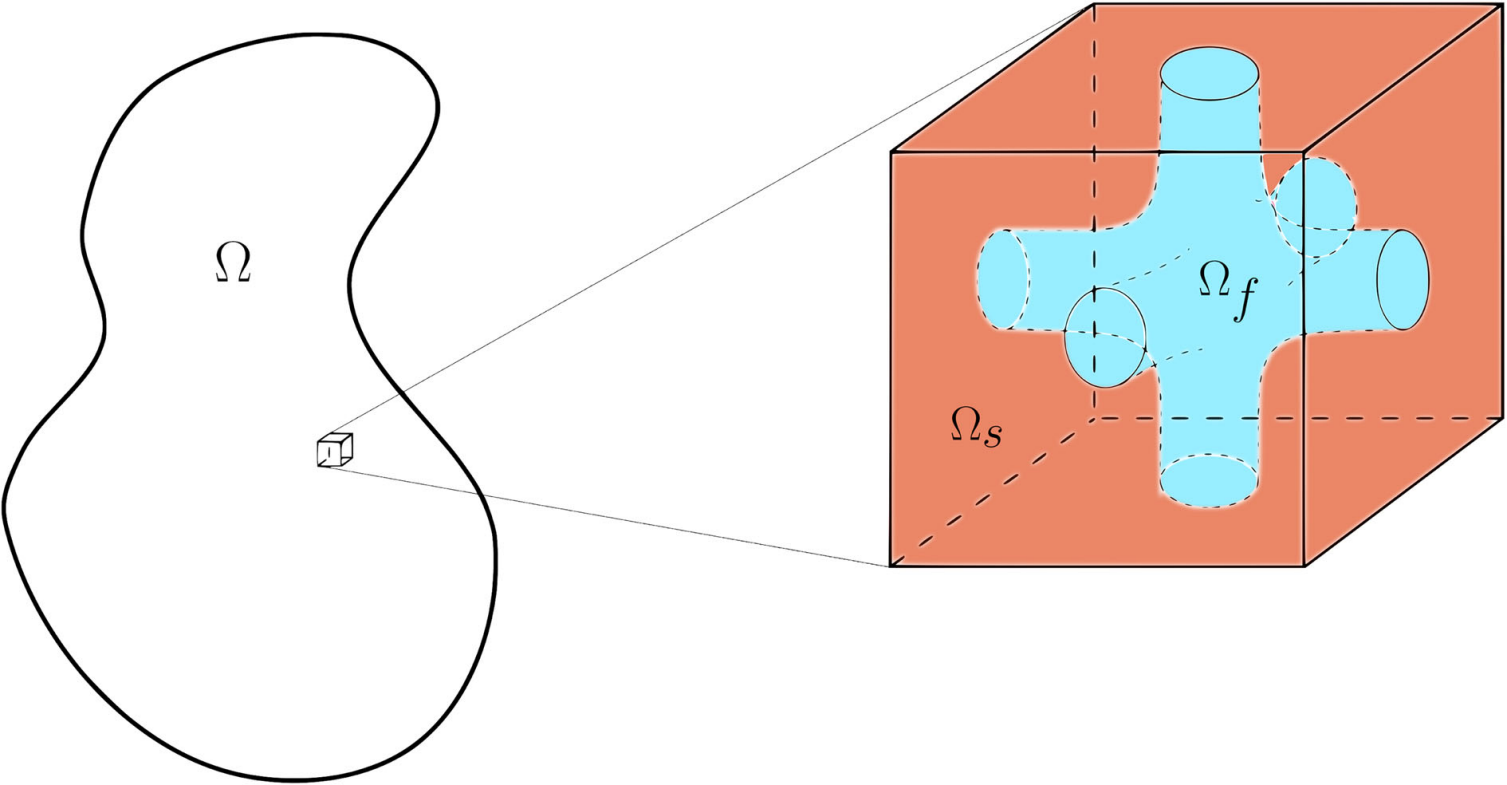
The domain $\Omega \subset \mathbb {R}^3$ with a zoomed in periodic cell representing the *pore scale*. In this case, the cell is represented by a cube comprising its elastic matrix portion $\Omega _s$ and the connected fluid compartment $\Omega _f$, here represented simply as three orthogonal smoothed cylinders.

#### Remark 4

(Pore scale Periodicity) The multiscale system of PDEs (28–33), supplemented by constitutive equations (34, 35), is formally defined on the whole pore scale spanned by variable ${\textbf{y}}$ with a parametric dependence on the macroscale ${\textbf{x}}$. However, the microstructure of porous materials is typically complex and comprises many features, so it is convenient to restrict our focus on a small portion of the pore scale structure. We then assume that our domain is made of repeated periodic cells as shown in Fig. 1, so that each field in (28–35) is $\textbf{y}$-periodic in order to restrict the analysis to the periodic cell. As such, we identify the domain $\Omega$ with its corresponding periodic cell, and similarly the porous matrix and the fluid phases constituting the cell are denoted by $\Omega _{s}$ and $\Omega _{f}$, respectively. The interface between the domains within the cell is then denoted as $\Gamma :=\partial \Omega _{s}\cap \partial \Omega _{f}$ , consistently with equation (10) with corresponding unit normal vector ${\textbf{n}}$ pointing outward from the fluid cell portion $\Omega _f$. This assumption is commonly adopted, as it improves and simplifies the presentation of the main results, and indeed ensures that the solution can be (usually numerically) obtained at a reduced computational cost. However, the macroscale results that we illustrate in the remainder of this work could be generalised to the case of ${\textbf{y}}$-boundedness, as performed in [12]. On the other hand, only the functional form of the macroscale model may be determined using the local boundedness approach. In the latter case, the model’s coefficients are associated with microscale problems that need formally to be resolved on the entire (unbounded as $\epsilon \rightarrow 0$) material microstructure, rather than a confined single portion. Furthermore, we could also assume variations of the periodic cell with respect to macroscale, but this is beyond the scope of this specific manuscript, as we highlight in the Remark below.

#### Remark 5

(Macroscopic uniformity) As is commonly known in multiscale materials, each macroscale point has an underlying microstructure that may well be different. This variation that can occur at each macroscale point has received much attention by a variety of approaches, see [12, 20, 29, 46, 47]. If we were to assume that the microscale could depend on the macroscale point, then this would add additional terms to our final model as we would need to carry out a proper application of the Reynolds’ transport theorem. This assumption, combined with the pore scale periodicity stated above, is equivalent to assuming that the geometry of the cell features a parametric dependence with respect to the macroscale ${\textbf{x}}$. This leads to considering more general situations, but in general entails a much greater computational cost, as one periodic cell per macroscale point ${\textbf{x}}$ would have to be considered. In this work, we focus on a more standard scenario by assuming that the porous structure does not depend on the variable $\textbf{x}$. This assumption, also referred to as *macroscopic uniformity*, allows for the following simple differentiation under the integral sign to be utilised, namely

37 $$\begin{aligned} \int \limits _{\Omega }\nabla _{{\textbf{x}}} \cdot (\cdot )\, \, d{{\textbf{y}}}=\nabla _{{\textbf{x}}}\cdot \int \limits _{\Omega }(\cdot )\, d{{\textbf{y}}}, \end{aligned}$$

where $(\cdot )$ is any tensor or vector quantity.

### The upscaling process

Each equation of the multiscale system of PDEs (28–33), as well as the multiscale constitutive relationships (34–35), can be formally viewed as the power series representation of an analytic function of $\epsilon$. Therefore, in view of the uniqueness of the power expansion of an analytic function, we obtain suitable sets of conditions by equating the coefficients multiplying $\epsilon ^l$ to zero for $l=0,1,...$. These conditions are then combined to obtain a macroscale closed system of PDEs in terms of the leading order fields $p^{(0)}_f$, $p^{(0)}_s$, ${\textbf{u}}^{(0)}$, ${\textbf{v}}^{(0)}$, ${\textsf{T}}^{(0)}_f$, ${\textsf{T}}^{(0)}_s$. The fields appearing in the power series expansions (36), including the leading order ones, are in principle functions of both the macroscale ${\textbf{x}}$ and the microscale ${\textbf{y}}$. As such, it is convenient to introduce the cell average operator defined by

38 $$\begin{aligned} \langle (\cdot ) \rangle _{\alpha }=\frac{1}{|\Omega |}\int \limits _{\Omega _\alpha }(\cdot )\, d{{\textbf{y}}} \quad \alpha =f,s, \end{aligned}$$

where integration is carried out over the representative fluid or solid portion of the periodic cell $\Omega$ with volume $|\Omega |$.

## Main results

Since we have now described all the relevant assumptions that should be applied in order to derive our model, we now can equate the coefficients of the powers of $\epsilon$ beginning with zero. This way we derive differential conditions that hold on the periodic cell spanned by the pore scale variable ${\textbf{y}}$, yet retaining a parametric dependence on the macroscale variable ${{\textbf {x}}}$.

Equating coefficients of $\epsilon ^0$ in (28–35) yields

39 $$\begin{aligned} \nabla _{{\textbf{y}}} \cdot {\textsf{T}}^{(0)}_s={\textbf{0}}&\qquad \qquad \text{ in } \quad \Omega _s \end{aligned}$$

40 $$\begin{aligned} \nabla _{{\textbf{y}}} \cdot {\textbf{u}}^{(0)}=0&\qquad \qquad \text{ in } \quad \Omega _s\end{aligned}$$

41 $$\begin{aligned} \nabla _{{\textbf{y}}} \cdot {\textsf{T}}^{(0)}_f={\textbf{0}}&\qquad \qquad \text{ in } \quad \Omega _f\end{aligned}$$

42 $$\begin{aligned} \nabla _{{\textbf{y}}} \cdot {\textbf{v}}^{(0)}=0&\qquad \qquad \text{ in } \quad \Omega _f\end{aligned}$$

43 $$\begin{aligned} \mathbf {\dot{u}}^{(0)}= {\textbf{v}}^{(0)}&\qquad \qquad \text{ on }\quad \Gamma \end{aligned}$$

44 $$\begin{aligned} {\textsf{T}}^{(0)}_s {\textbf{n}}={\textsf{T}}^{(0)}_f {\textbf{n}}&\qquad \qquad \text{ on }\quad \Gamma , \end{aligned}$$

supplemented by the following multiscale constitutive relationships

45 $$\begin{aligned} {\textsf{T}}^{(0)}_f= -p^{(0)}_f {\textsf{I}}&\qquad \qquad \text{ in } \quad \Omega _f\end{aligned}$$

46 $$\begin{aligned} 2 \bar{\mu }_s \xi _{{\textbf{y}}}{({\textbf{u}}^{(0)})}={\textsf{0}}&\qquad \qquad \text{ in }\quad \Omega _s. \end{aligned}$$

By equating the coefficients of $\epsilon ^1$ in (28–35), we obtain the following relationships

47 $$\begin{aligned} \nabla _{{\textbf{y}}} \cdot {\textsf{T}}^{(1)}_s +\nabla _{{\textbf{x}}} \cdot {\textsf{T}}^{(0)}_s={\textbf{0}}&\qquad \qquad \text{ in } \quad \Omega _s\end{aligned}$$

48 $$\begin{aligned} \nabla _{{\textbf{y}}} \cdot {\textbf{u}}^{(1)}+\nabla _{{\textbf{x}}} \cdot {\textbf{u}}^{(0)}=0&\qquad \qquad \text{ in } \quad \Omega _s\end{aligned}$$

49 $$\begin{aligned} \nabla _{{\textbf{y}}} \cdot {\textsf{T}}^{(1)}_f + \nabla _{{\textbf{x}}} \cdot {\textsf{T}}^{(0)}_f={\textbf{0}}&\qquad \qquad \text{ in } \quad \Omega _f\end{aligned}$$

50 $$\begin{aligned} \nabla _{{\textbf{y}}} \cdot {\textbf{v}}^{(1)}+\nabla _{{\textbf{x}}} \cdot {\textbf{v}}^{(0)}=0&\qquad \qquad \text{ in } \quad \Omega _f\end{aligned}$$

51 $$\begin{aligned} \dot{\textbf{u}}^{(1)}= {\textbf{v}}^{(1)}&\qquad \qquad \text{ on }\quad \Gamma \end{aligned}$$

52 $$\begin{aligned} {\textsf{T}}^{(1)}_s {\textbf{n}}={\textsf{T}}^{(1)}_f {\textbf{n}}&\qquad \qquad \text{ on }\quad \Gamma , \end{aligned}$$

supplemented by the following multiscale constitutive relationships

53 $$\begin{aligned} {\textsf{T}}^{(1)}_f= -p^{(1)}_f {\textsf{I}}+2 \bar{\mu }_f \xi _{{\textbf{y}}}{({\textbf{v}}^{(0)})} \,\,&\qquad \qquad \text{ in } \,\quad \Omega _f\end{aligned}$$

54 $$\begin{aligned} {\textsf{T}}^{(0)}_s=- p^{(0)}_s {\textsf{I}}+2 \bar{\mu }_s \xi _{{\textbf{y}}}{({\textbf{u}}^{(1)})}+2 \bar{\mu }_s \xi _{{\textbf{x}}}{({\textbf{u}}^{(0)})}\,\,&\qquad \qquad \text{ in }\,\quad \Omega _s. \end{aligned}$$

Substituting (45) into (41) we obtain that $\nabla _{{\textbf{y}}} p_f^{(0)}={\textbf{0}}$, yielding that $p_f^{(0)}$ does not depend on the microscale $\textbf{y}$, so

55 $$\begin{aligned} p_f^{(0)}=p_f^{(0)}(\textbf{x},t), \end{aligned}$$

i.e. the leading order pressure $p_f^{(0)}$ depends on the macroscale $\textbf{x}$ only. Equation (46) leads to $\textbf{u}^{(0)}=\boldsymbol{\omega } \wedge {\textbf{y}}+{\textbf{c}}$, that is, an infinitesimal rigid body motion where $\boldsymbol{\omega }$ and ${\textbf{c}}$ are ${\textbf{y}}$-constant vectors. However, since we are assuming $\textbf{y}$-periodicity, then $\boldsymbol{\omega }$ must reduce to zero and therefore ${\textbf{u}}^{(0)}={\textbf{c}}$, or equivalently

56 $$\begin{aligned} \textbf{u}^{(0)}=\textbf{u}^{(0)}(\textbf{x},t). \end{aligned}$$

### Macroscale Darcy’s law

This section is devoted to the analysis of the behaviour of the leading order fluid velocity. Substituting the constitutive relationships for the zeroth- and first-order fluid stress tensors (45) and (53) into the stress balance equation (49), accounting for the microscale incompressibility constraint (42) and interface conditions (43), we obtain the following Stokes’ boundary value problem for the variables ${\textbf{v}}^{(0)}$ and $p^{(1)}$ as follows

57 $$\begin{aligned} \nabla _{{\textbf{y}}}\cdot (2\bar{\mu }_f\xi _{{\textbf{y}}} ({\textbf{v}}^{(0)}))-\nabla _{{\textbf{y}}}p_f^{(1)}-\nabla _{{\textbf{x}}}p_f^{(0)}={\textbf{0}}&\qquad \qquad \,\, \text{ in } \quad \Omega _f\end{aligned}$$

58 $$\begin{aligned} \nabla _{{\textbf{y}}} \cdot {\textbf{v}}^{(0)}=0&\qquad \qquad \,\, \text{ in }\quad \Omega _f\end{aligned}$$

59 $$\begin{aligned} {\textbf{v}}^{(0)}=\dot{\textbf{u}}^{(0)}&\qquad \qquad \,\, \text{ on }\quad \Gamma , \end{aligned}$$

equipped with periodicity conditions on $\partial \Omega _f \setminus \Gamma$. The problem can be reformulated in terms of the *relative* leading order fluid velocity defined by

60 $$\begin{aligned} {\textbf{w}}(\textbf{x}, \textbf{y}, t)=\textbf{v}^{(0)}(\textbf{x}, \textbf{y}, t)-\dot{\textbf{u}}^{(0)}(\textbf{x}, t). \end{aligned}$$

Substituting (60) into the differential problem (57–59) and considering the macroscale only character of the leading order displacement as per (56), we obtain the following problem for (${\textbf{w}}$, $p_f^{(1)}$):

61 $$\begin{aligned} \nabla _{{\textbf{y}}}\cdot (2\bar{\mu }_f\xi _{{\textbf{y}}} ({\textbf{w}}))-\nabla _{{\textbf{y}}}p_f^{(1)}-\nabla _{{\textbf{x}}}p_f^{(0)}={\textbf{0}}&\qquad \qquad \, \text{ in }\quad \,\Omega _f,\end{aligned}$$

62 $$\begin{aligned} \nabla _{{\textbf{y}}} \cdot {\textbf{w}}=0&\qquad \qquad \,\, \text{ in }\quad \, \Omega _f,\end{aligned}$$

63 $$\begin{aligned} {\textbf{w}}={\textbf{0}}&\qquad \qquad \,\, \text{ on }\quad \,\, \Gamma , \end{aligned}$$

and again periodicity conditions on $\partial \Omega _f \setminus \Gamma$.

We now observe that the linear system of PDEs (61–63) has non-trivial solutions when $\nabla _{{\textbf{x}}} p^{(0)} \ne {\textbf{0}}$, hence we state the solution (${\textbf{w}}$, $p_f^{(1)}$) in terms of the following ansatz

64 $$\begin{aligned} {\textbf{w}}=-{\textsf{W}}\nabla _{{\textbf{x}}} p_f^{(0)}; \quad p_f^{(1)}=-{\textbf{p}}\cdot \nabla _{{\textbf{x}}} p_f^{(0)}+d, \end{aligned}$$

where $p^{(1)}_f$ can only be specified up to an arbitrary $\textbf{y}$-constant function *d*. Equation (64) is the solution of the problem (61-63) provided that the auxiliary second rank tensor $\textsf{W}$ and vector $\textbf{p}$ satisfy the following cell problem

65a $$\begin{aligned} \nabla _{{\textbf{y}}} \cdot (2 \bar{\mu }_f \hat{\xi }_{{\textbf{y}}}({{\textsf{W}}}))-(\nabla _{{\textbf{y}}} \textbf{p})^T +{\textsf{I}}={\textsf{0}}&\qquad \qquad \text{ in } \quad \Omega _f \end{aligned}$$

65b $$\begin{aligned} \nabla _{{\textbf{y}}} \cdot {{\textsf{W}}}^{\mathrm T}=\textbf{0}&\qquad \qquad \text{ in } \quad \Omega _f \end{aligned}$$

65c $$\begin{aligned} {\textsf{W}}={\textsf{0}}&\qquad \qquad \text{ on } \quad \Gamma , \end{aligned}$$

where once again periodic conditions apply to the boundary $\partial \Omega _f \setminus \Gamma$ and a further condition is to be imposed on ${\textbf{p}}$ for the solution to be unique (e.g. zero average on periodic cell), and the third rank tensor $\hat{\xi }_{{\textbf{y}}}({{\textsf{W}}})$ is such that its component-wise representation reads:

66 $$\begin{aligned} {[}\hat{\xi }_{{\textbf{y}}}({{\textsf{W}}}))]_{ijk}=\frac{\partial {\textsf{W}}_{ik}}{\partial y_j}+\frac{\partial {\textsf{W}}_{jk}}{\partial y_i}, \end{aligned}$$

see also Appendix A for a complete component-wise representation of the problem.

Taking the integral average of (64) over the fluid domain leads to

67 $$\begin{aligned} \langle \textbf{w}\rangle _f=-\langle {{\textsf{W}}}\rangle _f\nabla _{{\textbf{x}}}p_f^{(0)}, \end{aligned}$$

i.e. the average relative leading order fluid velocity is described by Darcy’s law.

### Poroelasticity on the macroscale

This section is focussed on the derivation of the macroscale stress balance equations in terms of the leading order elastic displacement and fluid and solid pressures, that is ${\textbf{u}}^{(0)}$, $p_f^{(0)}$, and $p_s^{(0)}$, respectively. We start by adding up the periodic cell averages of Eqs. (47) and (49) over $\Omega _s$ and $\Omega _f$, respectively, obtaining

68 $$\begin{aligned} \begin{aligned} \frac{1}{|\Omega |}\bigg (\int \limits _{\Omega _s} \nabla _{{\textbf{y}}}\cdot {\textsf{T}}^{(1)}_s\,d{{\textbf{y}}} + \int \limits _{\Omega _f}\nabla _{{\textbf{y}}}\cdot {\textsf{T}}^{(1)}_f\,d{{\textbf{y}}} + \int \limits _{\Omega _s} \nabla _{{\textbf{x}}} \cdot {\textsf{T}}^{(0)}_s\,d{{\textbf{y}}} + \int \limits _{\Omega _f} \nabla _{{\textbf{x}}}\cdot {\textsf{T}}^{(0)}_f\,d{{\textbf{y}}}\bigg )=0. \end{aligned} \end{aligned}$$

Applying the divergence theorem with respect to $\textbf{y}$ to the first two integrals and rearranging the last two terms by enforcing the assumption of macroscopic uniformity (37), we obtain

69 $$\begin{aligned} \begin{aligned} \frac{1}{|\Omega |}\bigg (\int \limits _{\partial \Omega _s\backslash \Gamma } {\textsf{T}}^{(1)}_s\textbf{n}_{\Omega _s}\,\textrm{dS } {-}\int \limits _{\Gamma } {\textsf{T}}^{(1)}_s\textbf{n}\, \textrm{dS} {+}\int \limits _{\partial \Omega _f\backslash \Gamma }{\textsf{T}}_f^{(1)}  \textbf{n}_{\Omega _f}\,\textrm{dS} {+}\int \limits _{\Gamma }{\textsf{T}}^{(1)}_f\textbf{n}\,\textrm{dS}{+} \nabla _{{\textbf{x}}} \cdot \int \limits _{\Omega _s} {\textsf{T}}^{(0)}_s\,d{{\textbf{y}}} {+}\nabla _{{\textbf{x}}}\cdot \int \limits _{\Omega _f} {\textsf{T}}^{(0)}_f\,d{{\textbf{y}}}\bigg ){=}0, \end{aligned} \end{aligned}$$

where $\textbf{n}$ is the unit normal vector to $\Gamma$ pointing out of the fluid domain $\Omega _f$, which means that the unit vector normal to $\Gamma$ which is pointing outward with respect to the solid domain $\Omega _s$ is given by $-\textbf{n}$. We also denote by $\textbf{n}_{\Omega _s}$ and $\textbf{n}_{\Omega _f}$ the unit outward normal vectors to the $\partial \Omega _s\backslash \Gamma$ and $\partial \Omega _f\backslash \Gamma$ cell boundaries, respectively.

As the surface integrals over the boundaries $\partial \Omega _s \backslash \Gamma$ and $\partial \Omega _f \backslash \Gamma$ reduce to zero by enforcing $\textbf{y}$-periodicity, then relationship (69) reduces to

70 $$\begin{aligned} \begin{aligned} \frac{1}{|\Omega |}\bigg (-\int \limits _{\Gamma } {\textsf{T}}^{(1)}_s\textbf{n}\,\text{ dS } + \int \limits _{\Gamma }{\textsf{T}}^{(1)}_f\textbf{n} \,\text{ dS }+\nabla _{{\textbf{x}}} \cdot \int \limits _{\Omega _s} {\textsf{T}}^{(0)}_s\,d{{\textbf{y}}} +\nabla _{{\textbf{x}}}\cdot \int \limits _{\Omega _f} {\textsf{T}}^{(0)}_f\,d{{\textbf{y}}}\bigg )=0. \end{aligned} \end{aligned}$$

By exploiting continuity of the order one tractions (52), the contributions over the interface $\Gamma$ vanish, leading to

71 $$\begin{aligned} \nabla _{{\textbf{x}}} \cdot \langle {\textsf{T}}_s^{(0)}\rangle _s +\nabla _{{\textbf{x}}} \cdot \langle {\textsf{T}}_f^{(0)}\rangle _f={\textbf{0}}, \end{aligned}$$

which can be rewritten by considering the leading order constitutive relationship for the fluid stress tensor (45) as

72 $$\begin{aligned} \nabla _{{\textbf{x}}} \cdot \tilde{{\textsf{T}}}={\textbf{0}}, \end{aligned}$$

where we have defined the effective macroscale stress tensor as

73 $$\begin{aligned} \tilde{{\textsf{T}}}=\langle {\textsf{T}}_s^{(0)}\rangle _s-\varphi _f p_f^{(0)} {\textsf{I}}. \end{aligned}$$

We recall that the fluid pressure $p_f^{(0)}({\textbf{x}},t)$ is constant with respect to the microscale $\textbf{y}$ (cf. Eq. (55)), and $\varphi _f:=|\Omega _f|/|\Omega |$ is the fluid volume fraction, i.e. the *porosity* of the system.

In the remainder of this section, we investigate how to exploit the relationship obtained by equating the coefficients of the same power of $\epsilon$ in order to determine the leading order elastic stress tensor ${\textsf{T}}_s^{(0)}$ as a function of zeroth-order displacement and pressure fields.

We commence by substituting the constitutive equation for the leading order solid stress tensor (54) into both the zeroth-order solid stress balance and the continuity of tractions (39) and (44), respectively. This way, by considering the non-divergence-free constraint (48) and the leading order constitutive equation for the fluid stress tensor (45), we obtain the following ${\textbf{y}}$-periodic problem for the order one displacement vector ${\textbf{u}}^{(1)}$ and the leading order solid pressure $p_s^{(0)}$, as follows

74 $$\begin{aligned}  &   \nabla _{{\textbf{y}}}\cdot (2\bar{\mu }_s\xi _{{\textbf{y}}} ({\textbf{u}}^{(1)}))-\nabla _{{\textbf{y}}} p_s^{(0)}= -\nabla _{{\textbf{y}}}\cdot (2\bar{\mu }_s\xi _{{\textbf{x}}} ({\textbf{u}}^{(0)})) \quad \,\, \,\, \text{ in } \quad \Omega _s,\end{aligned}$$

75 $$\begin{aligned}  &   \nabla _{{\textbf{y}}} \cdot {\textbf{u}}^{(1)}=-\nabla _{{\textbf{x}}} \cdot {\textbf{u}}^{(0)}\,\,\,\qquad \qquad \qquad \qquad \qquad \qquad \,\,\,\quad \text{ in } \quad \Omega _s,\end{aligned}$$

76 $$\begin{aligned}  &   \left( 2 \bar{\mu }_s \xi _{{\textbf{y}}}{({\textbf{u}}^{(1)})}- p^{(0)}_s {\textsf{I}}\right) {\textbf{n}}= -\left( 2 \bar{\mu }_s \xi _{{\textbf{x}}}{({\textbf{u}}^{(0)})}+p_f^{(0)} {\textsf{I}}\right) {\textbf{n}} \,\,\quad \text{ on } \quad \Gamma , \end{aligned}$$

together with $\textbf{y}$-periodicity on $\partial \Omega _s \setminus \Gamma$.

The solution $({\textbf{u}}^{(1)}, p^{(0)}_s)$ of the problem given by equations (74–76), exploiting linearity, is given by

77 $$\begin{aligned} \textbf{u}^{(1)}= &   \mathcal {R} {:}\xi _\mathbf{{x}}(\textbf{u}^{(0)})+ \textbf{a}p_f^{(0)}+\textbf{c}(\textbf{x}),\end{aligned}$$

78 $$\begin{aligned} p_s^{(0)}= &   {\textsf{S}}{:}\xi _\mathbf{{x}}(\textbf{u}^{(0)})+ \phi p_f^{(0)}, \end{aligned}$$

where $\textbf{c}$ is an arbitrary $\textbf{y}$-constant vector-valued function, $\mathcal {R}$ is a third rank tensor and ${\textsf{S}}$ is a second rank tensor. The ansätze (77-78) represent the solution of the problem provided that $(\mathcal {R}, {\textsf{S}})$ and $(\textbf{a}, \phi )$ solve the following cell problems

79a $$\begin{aligned}  &   \nabla _\mathbf{{y}} \cdot (2\bar{\mu }_s\tilde{\xi }_\mathbf{{y}} [\mathcal {R}])-\nabla _\mathbf{{y}} \cdot ({\textsf{I}} \otimes {\textsf{S}})=-\nabla _\mathbf{{y}} \cdot (2\bar{\mu }_s\mathbb {I}^{S}) \quad \,\, \text{ in }\quad \,\, \Omega _s \end{aligned}$$

79b $$\begin{aligned}  &   \nabla _\mathbf{{y}} \cdot \mathcal {R}=-{\textsf{I}} \qquad \qquad \qquad \qquad \qquad \qquad \,\,\qquad \qquad \text{ in }\quad \,\, \Omega _s, \end{aligned}$$

79c $$\begin{aligned}  &   \left( 2\bar{\mu }_s \tilde{\xi }_\mathbf{{y}} [\mathcal {R}]-{\textsf{I}} \otimes \textsf{S}\right) {\textbf{n}}=-2\bar{\mu }_s \mathbb {I}^{S} \mathbf{{n}} \qquad \qquad \qquad \,\quad \text{ on } \quad \Gamma , \end{aligned}$$

and

80a $$\begin{aligned}  &   \nabla _\mathbf{{y}} \cdot (2\bar{\mu }_s\xi _\mathbf{{y}} (\textbf{a}))-\nabla _\mathbf{{y}} \phi =\textbf{0}&\quad \text{ in } \quad \Omega _s, \end{aligned}$$

80b $$\begin{aligned}  &   \nabla _\mathbf{{y}} \cdot \mathbf{{a}} =0&\quad \text{ in }\quad \Omega _s \end{aligned}$$

80c $$\begin{aligned}  &   \left( -\phi \textsf{I}+2\bar{\mu }_s \xi _\mathbf{{y}} (\textbf{a}) \right) \textbf{n}=-\textbf{n}&\quad \text{ on } \quad \Gamma , \end{aligned}$$

where the problems (79a–79c) and (80a–80c) are to be supplemented by periodic conditions on $\partial \Omega _s\setminus \Gamma$ and we require one further condition on $\mathcal {R}$ and **a** ensure uniqueness, e.g. prescribing their cell average equal to a chosen constant, such as zero. The fourth rank tensor $\tilde{\xi }_\mathbf{{y}} [\mathcal {R}]$ has the following component-wise representation

81 $$\begin{aligned} \tilde{\xi }_\mathbf{{y}} [\mathcal {R}]_{ijkl}=\frac{1}{2}\left( \frac{\partial R_{ikl}}{\partial y_j}+\frac{\partial R_{jkl}}{\partial y_i} \right) . \end{aligned}$$

The divergence operator in problem (79a–79c) acts on the second component of the fourth rank tensors in (79a) (and so does the corresponding contraction between them and the unit normal in (79c)), and on the first component of the third rank tensor in (79b). A comprehensive representation of the ansätze and the cell problems in components is provided in Appendix A.

Given that the order one displacement $\textbf{u}^{(1)}$ and the leading order solid pressure $p_s^{(0)}$ can be written as functions of the leading order displacement ${\textbf {u}}^{(0)}$ and fluid pressure $p^{(0)}_f$, by substituting the ansätze (77–78) in the leading order constitutive relationship for the solid stress tensor ${\textsf{T}}^{(0)}_s$ 35, we obtain

82 $$\begin{aligned} {\textsf{T}}_s^{(0)}=\left( \mathbb {C}+\mathbb {C:N}-{\textsf{I}} \otimes {\textsf{S}}\right) :\xi _\mathbf{{x}}(\textbf{u}^{(0)}) +\left( \mathbb {C}:{\textsf{P}}-\phi {\textsf{I}}\right) p_f^{(0)} \end{aligned}$$

and the fourth rank tensors $\mathbb {C}$ and $\mathbb {N}$ and second rank tensor ${\textsf{P}}$ are defined as

83 $$\begin{aligned} \mathbb {N}=\tilde{\xi }_\mathbf{{y}} [\mathcal {R}], \qquad \mathbb {C}=2 \bar{\mu }_s \mathbb {I}^{S}, \qquad {\textsf{P}}=\xi _\mathbf{{y}}(\textbf{a}). \end{aligned}$$

In order to determine the overall constitutive equation of the material, we first average equation (82) over the solid portion of the domain, which yields

84 $$\begin{aligned} \langle {\textsf{T}}_{s}^{(0)}\rangle _s= \tilde{\mathbb {C}}:\xi _{{\textbf{x}}}(\textbf{u}^{(0)})+\langle \mathbb {C}:{\textsf{P}}-\phi {\textsf{I}}\rangle _s p_f^{(0)}, \end{aligned}$$

where the homogenised elasticity tensor $\tilde{\mathbb {C}}$ is defined as

85 $$\begin{aligned} \tilde{\mathbb {C}}=\langle \mathbb {C}+\mathbb {C:N}-{\textsf{I}} \otimes {\textsf{S}} \rangle _s. \end{aligned}$$

Therefore, the overall stiffness tensor $\tilde{{\textsf{T}}}$ (cf. relationship (73)) satisfying the macroscale balance equation (72) reads

86 $$\begin{aligned} \tilde{{\textsf{T}}}=\tilde{\mathbb {C}}:\xi _{{\textbf{x}}}(\textbf{u}^{(0)})+(\langle \mathbb {C}:{\textsf{P}}-\phi {\textsf{I}}\rangle _s-\varphi _f \textsf{I}) p^{(0)}_f. \end{aligned}$$

Equation (72), supplemented by constitutive equation (86), represents the macroscale momentum balance for the whole system and describes the overall behaviour of the poroelastic material in terms of the leading order elastic displacement ${\textbf{u}}^{(0)}$ and fluid pressure $p^{(0)}_f$. We now summarise our findings and derive the final equation which shows that the classical mass conservation constraint arising from the interplay between intrinsically incompressible solid and fluid phases is obtained as a result of our formulation.

### Solid volume and fluid exchange balance

We have now obtained Darcy’s law (67), and have shown that the constitutive equation for the poroelastic material (86) reads as an additive decomposition of an elastic component, which is related to the macroscale strains via the drained elasticity tensor (85), and a contribution proportional to the fluid pressure, consistently with classical results. The system of equations for ${\textbf{u}}^{(0)}$ and $p^{(0)}_f$ is then typically closed by a condition that relates variations of the fluid pressure with variations of the solid volume and the fluid exchanged. In the context of the classical theory of poroelasticity, such relationship can be stated as (in a notation relatable to that of this manuscript, and for a macroscopically isotropic porous medium), for compressible constituents, as

87 $$\begin{aligned} \dot{{\tilde{p}}}=- M (\alpha \nabla \cdot \dot{\tilde{{\textbf{u}}}} + \nabla \cdot {\textbf{q}}), \end{aligned}$$

where $\tilde{{\textbf{u}}}$ represents the poroelastic displacement, ${\tilde{p}}$ the poroelastic fluid pressure and ${\textbf{q}}$ is the so called discharge flow vector, which describes the averaged flow of the fluid relative to the solid motion and is the vector field satisfying Darcy’s law.^1^ The parameters *M* and $\alpha$ are Biot’s modulus and Biot’s coefficient. They physically represent the resistance of the poroelastic medium to volume changes for a given pressure change (at constant strains), and the ratio of fluid gained (or lost) to solid volume changes. The limit for intrinsically incompressible constituent, that is, the equivalency between the change in solid volume and the volume of fluid exchanged, is achieved by taking the limit as $M \rightarrow +\infty$ and $\alpha =1$, which indeed leads to $\nabla \cdot \dot{\tilde{{\textbf{u}}}} + \nabla \cdot {\textbf{q}}=0$. We now prove that our model satisfies this condition. In particular, the poroelastic displacement and discharge flow vector are to be identified with the leading order displacement ${\textbf{u}}^{(0)}$ and the average of the relative leading order fluid velocity $\left\langle {\textbf{w}} \right\rangle _f$. The claim we need to prove then reads as follows

88 $$\begin{aligned} \nabla _{{\textbf{x}}} \cdot \dot{{\textbf{u}}}^{(0)} + \nabla _{{\textbf{x}}} \cdot \left\langle {\textbf{w}} \right\rangle _f=0. \end{aligned}$$

#### Remark 6

(Proof of claim (88)) We commence by applying the periodic cell average operator (38) to the order one constraint (50) over the fluid portion of the domain $\Omega _f$ to obtain

89 $$\begin{aligned} \nabla _{{\textbf{x}}} \cdot \langle \textbf{v}^{(0)}\rangle _f+\frac{1}{|\Omega |}\int \limits _{\Gamma } {\textbf{v}}^{(1)}\cdot {\textbf{n}}\, \text {dS}=0, \end{aligned}$$

where we have applied the divergence theorem with respect to the microscale variable ${\textbf{y}}$ as well as ${\textbf{y}}$-periodicity to neglect the contribution over the period cell boundary $\partial \Omega _f\setminus \Gamma$ and macroscopic uniformity (37).

Similarly, we can apply the cell average operator (38) to the order one constraint (48) over the solid portion of the domain $\Omega _s$ to obtain

90 $$\begin{aligned} \varphi _s\nabla _{{\textbf{x}}} \cdot \textbf{u}^{(0)}+\frac{1}{|\Omega |}\int \limits _{\Gamma } {\textbf{u}}^{(1)}\cdot {\textbf{n}}_s\, \text {dS}=0, \end{aligned}$$

having applied again the divergence theorem with respect to ${\textbf{y}}$ and ${\textbf{y}}$-periodicity so that the contributions over the periodic boundary $\partial \Omega _s \setminus \Gamma$ reduce to zero. Here we have also considered that the leading order displacement ${\textbf{u}}^{(0)}$ does not depend on the microscale ${\textbf{y}}$ and $\varphi _s:=|\Omega _s|/|\Omega |$ is the solid volume fraction. Since the unit normal ${\textbf{n}}$ is assumed to be pointed outward with respect to the fluid domain, we also have ${\textbf{n}}_s=-{\textbf{n}}$, so we can also rewrite Eq. (90) as

91 $$\begin{aligned} \varphi _s\nabla _{{\textbf{x}}} \cdot \textbf{u}^{(0)}-\frac{1}{|\Omega |}\int \limits _{\Gamma } {\textbf{u}}^{(1)}\cdot {\textbf{n}}\, \text {dS}=0. \end{aligned}$$

As the microscale cell is not varying in time, by taking the time derivative of relationship (91) we also obtain

92 $$\begin{aligned} \varphi _s\nabla _{{\textbf{x}}} \cdot \dot{{\textbf{u}}}^{(0)}-\frac{1}{|\Omega |}\int \limits _{\Gamma } \dot{{\textbf{u}}}^{(1)}\cdot {\textbf{n}}\, \text {dS}=0. \end{aligned}$$

By summing up relationships (92) and (89), we finally obtain

93 $$\begin{aligned} \varphi _s\nabla _{{\textbf{x}}} \cdot \dot{{\textbf{u}}}^{(0)}+\nabla _{{\textbf{x}}} \cdot \langle \textbf{v}^{(0)}\rangle _f=0, \end{aligned}$$

having applied continuity of velocities on the fluid–solid interface (51), which in particular also implies continuity of the corresponding normal components. By using the definition of relative velocity ${\textbf{w}}$ (60), we can rewrite the constraint (93) as

94 $$\begin{aligned} \varphi _s\nabla _{{\textbf{x}}} \cdot \dot{{\textbf{u}}}^{(0)}+\nabla _{{\textbf{x}}} \cdot \langle {\textbf{w}}+\mathbf {\dot{u}}^{(0)}\rangle _f=0, \end{aligned}$$

and since the leading order displacement does not depend on ${\textbf{y}}$, we have $\langle {\textbf{w}}+\mathbf {\dot{u}}^{(0)}\rangle _f=\langle {\textbf{w}}\rangle _f+\varphi _f \dot{{\textbf{u}}}^{(0)}$, which leads to

95 $$\begin{aligned} \nabla _{{\textbf{x}}} \cdot \dot{{\textbf{u}}}^{(0)}+\nabla _{{\textbf{x}}} \cdot \langle {\textbf{w}}\rangle _f=0, \end{aligned}$$

as $$\varphi _f+\varphi _s=1$$. $$\Box $$

We have now derived all the equations required to be able to state our macroscale model. The equations in the macroscale model describe the effective poroelastic behaviour of the material relating to the pressure, the average fluid velocity and the elastic displacement. Therefore, the macroscale model to be solved is given by

96a $$\begin{aligned}&\langle \textbf{w}\rangle _f=-\langle W\rangle _f\nabla _{{\textbf{x}}}p^{(0)}, \end{aligned}$$

96b $$\begin{aligned}&\nabla _{{\textbf{x}}} \cdot \tilde{{\textsf{T}}}={\textbf{0}}, \end{aligned}$$

96c $$\begin{aligned}&\tilde{{\textsf{T}}}=\tilde{\mathbb {C}}\xi _{{\textbf{x}}}(\textbf{u}^{(0)})+(\langle \mathbb {C}:{\textsf{P}}-\phi {\textsf{I}}\rangle _s-\varphi _f \textsf{I}) p^{(0)}_f, \end{aligned}$$

96d $$\begin{aligned}&\nabla _{{\textbf{x}}} \cdot \dot{{\textbf{u}}}^{(0)}+\nabla _{{\textbf{x}}} \cdot \langle {\textbf{w}}\rangle _f=0, \end{aligned}$$

and represents a closed system of PDEs (given Darcy’s law) subjected to appropriate macroscale boundary conditions.

The major difference between the current formulation and standard approaches which start from compressible constituents resides in (a) the coefficients of the model, which are to be computed by solving a different set of periodic cell problems, which require less input parameters (in particular, in the isotropic case, only the shear modulus needs to be specified for the solid matrix), and (b) the present formulation leads to the balance (88) without enforcing a posteriori assumption on derived coefficients based on their physical meaning in the case of intrinsic incompressibility. In fact, standard approaches which are utilised to derive poroelasticity from the microstructure lead to a poroelastic system of equations which is analogous to (96), but with Eq. (88) replaced by the balance equation (87). However, Biot’s modulus and coefficients are in this case to be computed by solving corresponding cell problems (see for example [12, 52]), and enforcing strict incompressibility via limit values of the parameters (say Poisson’s ratio approaching 0.5 in the case of isotropy) does not lead to a rigorous proof that the solution of such cell problems leads to the limits $M \rightarrow +\infty$ and $\alpha =1$, thus leading to (88). Instead, incompressible poroelasticity derived from the microstructure featuring the constraint (88) can be derived by starting from strict incompressibility from the commencement, which is the approach we embrace in this work. We further note that, if needed, the solid pressure can also be retrieved via (78), although, as expected, this is ultimately not needed to close the problem for the leading order displacement and fluid pressure, consistently with the standard theory of poroelasticity.

## Conclusion

We have presented a new formulation to derive the equations of poroelasticity from a microstructure characterised by intrinsically incompressible constituents at the scale of the pores. We have started from the fluid–structure interaction problem between a linear elastic isotropic porous matrix and an incompressible Newtonian fluid (by neglecting inertia and body forces), and by enforcing incompressibility by considering both the fluid and solid pressures, which formally read as the Lagrangian multipliers associated with the incompressibility constraints for both phases. The coupling between phases, enforced via continuity of velocities and tractions, occurs between two systems of linear saddle point differential problems, where both the fluid viscosity and the elastic shear modulus are assumed to be strictly positive, heterogeneous functions. We have then performed the upscaling of the resulting coupled equations by means of the asymptotic (periodic) homogenisation method. The main result consists of a macroscale system of Biot’s type equations which automatically satisfies the condition that is to be met at the macroscale when both phases are individually incompressible at the pore scale, i.e. variations of the solid volume are identically balanced by variations of the fluid content. In particular, while the obtained hydraulic conductivity which appears in the obtained Darcy’s law is the natural generalisation of previous results [52] for heterogeneous viscosities, the effective stiffness tensor is to be computed by solving non-standard pore scale cell problems specific to this new formulation. The latter have a saddle point linear structure, equipped with corresponding auxiliary pressures and non-divergence-free constraints in addition to the standard auxiliary volume and boundary load related to the matrix stiffness (in this case, solely the shear modulus as we are assuming isotropy of the solid phase), its variations and the pore scale geometry. The benefit of this new formulation is that intrinsic incompressibility is considered from the commencement, which means that the macroscale equations that are expected to hold in that limit are automatically obtained as a result of the upscaling process, which does not happen when embracing the traditional derivation. In this latter case, the results hold for compressibility of the solid matrix, and the macroscale system of PDEs reduces to one meeting the above-mentioned condition only when approaching physically appropriate limit regimes which are based on physically intuitive arguments and do not rigorously follow from the definition of the effective parameters formulated in terms of the underlying pore structure. In other words, although there exist results based on numerical simulations which are reassuring on the reliability of the classical formulation when approaching the solid matrix incompressibility, e.g. [22], the traditional formulations are not amenable of the strict incompressibility limit, which is indeed the novelty of this work. This manuscript is open to several improvements. First of all, the formulation has been derived by embracing a number of simplifying modelling assumptions such as neglecting inertia, body forces and material anisotropy. The formulation can be extended in a straightforward way (in a small deformations regime) to linearised inertia [23], as done in [12]; however, the extension, though possible, is less trivial if a full representation of the fields, rather than a single fixed frequency approach is embraced, as shown in [27]. An extension to generic body forces is also possible and would neither alter the structure of the pore scale problems nor modify the obtained constitutive relationship unless more complex representations featuring Helmholtz-type representation of such forces [53], or pre-stress is involved, see, e.g. [25]. Anisotropy of the elastic matrix could also be considered as per Remark 1. For instance, in [26], the Authors investigate the properties of the anisotropic, linear elastic, stiffness tensor and its restrictions by extending the analysis also to non-standard metrics.

Further development of this work will involve implementation of the model (by either semi-analytical or numerical means) and the solutions should then be compared with those recently obtained for example in [22]. The comparison will be illuminating especially to evaluate the discrepancy between results obtained by applying standard formulations for values of the solid matrix Poisson’s ratio close to 0.5 against those obtained via the present work, where the solid matrix is strictly incompressible. The results are open for further theoretical extensions as in particular the saddle point formulation could be extended also to compressible materials (see, e.g. [31]), so that this work could pave the way for a unified poroelastic theory that could automatically incorporate incompressibility of the individual phases, which is currently not the case for standard approaches such as [12]. Finally, the solutions of this model and/or its extension are applicable to incompressible elastic materials, that are for instance encountered in real-world scenarios of interest, e.g. biological tissues such as the lungs, which feature the interplay between an incompressible elastic matrix and a compressible fluid, that is, air flowing through the airways [6, 16, 19, 32].

## Data Availability

There are no additional data supporting this study.

## The component-wise pore scale PDEs

In this section, we write the pore scale cell problems that we obtained in the main body of the manuscript involving the third and fourth rank tensors defined in (66) and (81), respectively, by representing them in components by means of the Einstein convention on repeated indices. The problem (61–63) that holds for (${\textbf{w}}$, $p_f^{(0)}$) in the fluid compartment, component-wise, reads:

97 $$\begin{aligned}&\frac{\partial }{\partial y_j} \left( \bar{\mu }_f \left( \frac{\partial w_{i}}{\partial y_j}+{\frac{\partial w_{j}}{\partial y_i}}\right) \right) -\frac{\partial p^{(1)}_f}{\partial y_i}- \frac{\partial p^{(0)}_f}{\partial x_i}=0\, \quad \text{ in } \quad \Omega _f \end{aligned}$$

98 $$\begin{aligned}&\frac{\partial w_{i}}{\partial y_i}=0 \qquad \qquad \qquad \qquad \qquad \qquad \qquad \qquad \qquad \text{ in } \quad \Omega _f&\end{aligned}$$

99 $$\begin{aligned}&w_i=0\qquad \qquad \qquad \qquad \qquad \qquad \qquad \qquad \qquad \quad \text{ on } \quad \Gamma . \end{aligned}$$

Given the ansatz (64), we have $w_i=-W_{ik}\frac{\partial p^{(0)}}{\partial x_k}$, $w_j=-W_{jk}\frac{\partial p^{(0)}}{\partial x_k}$, $p_f^{(1)}=-p_k \frac{\partial p^{(0)}}{\partial x_k}$ so that the cell problem for the second rank tensor ${\textsf{W}}$ in components reads, since $\frac{\partial p^{(0)}_f}{\partial x_i}=\delta _{ik}\frac{\partial p^{(0)}_f}{\partial x_k}$:

100 $$\begin{aligned}&\frac{\partial }{\partial y_j} \left( \bar{\mu }_f \left( \frac{\partial {\textsf{W}}_{ik}}{\partial y_j}+{\frac{\partial {\textsf{W}}_{jk}}{\partial y_i}}\right) \right) -\frac{\partial p_k}{\partial y_i}+ \delta _{k}=0 \quad \text{ in } \quad \Omega _f \end{aligned}$$

101 $$\begin{aligned}&\frac{\partial {\textsf{W}}_{ik}}{\partial y_i}=0 \qquad \qquad \qquad \qquad \qquad \qquad \qquad \qquad \quad \text{ in } \quad \Omega _f&\end{aligned}$$

102 $$\begin{aligned}&{\textsf{W}}_{ik}=0\qquad \qquad \qquad \qquad \qquad \qquad \qquad \qquad \qquad \text{ on } \quad \Gamma . \end{aligned}$$

We notice that the above problem generalises the standard Stokes’-type problem arising from homogenisation of the Stokes’ problem for an heterogeneous viscosity and, when the latter is a constant, since (101) holds, the problem reduces to the classical one for example investigated in [48]. From the component-wise representation (100–102), we can also deduce that the problem (65a–65c) corresponds to three vector-valued problems driven by volume loads corresponding to the unit vectors in the three orthogonal directions, for $k=1,2$, and 3, as also illustrated in [48, 50].

The problem (74–76) that holds in the solid compartment $\Omega _s$ for the fields $({\textbf{u}}^{(1)}, p^{(0)}_s)$ can be rewritten in components as

103 $$\begin{aligned}  &   \frac{\partial }{\partial y_j} \left( \bar{\mu }_s \left( \frac{\partial u_i^{(1)}}{\partial y_j} +\frac{\partial u_j^{(1)}}{\partial y_i}\right) \right) -\frac{\partial (p^{(0)}_s \delta _{ij})}{\partial y_i}=-\frac{\partial (2 \bar{\mu }_s \xi _{ij})}{\partial x_j},\end{aligned}$$

104 $$\begin{aligned}  &   \frac{\partial u_i^{(1)}}{\partial y_i}=-\frac{\partial u_i^{(0)}}{\partial x_i}, \end{aligned}$$

supplemented by periodic conditions on $\partial \Omega _s {\setminus } \Gamma$ and by the following conditions on the interface $\Gamma$

105 $$\begin{aligned} \left( \bar{\mu }_s \left( \frac{\partial u_i^{(1)}}{\partial y_j}+\frac{\partial u_j^{(1)}}{\partial y_i}\right) -p^{(0)}_s \delta _{ij} \right) n_j=-(2 \bar{\mu }_s \xi _{ij}+p^{(0)}_f \delta _{ij}) n_j, \end{aligned}$$

where we defined $\xi _{ij}=\frac{1}{2}\left( \frac{\partial u_i^{(0)}}{\partial x_j}+\frac{\partial u_j^{(0)}}{\partial x_i}\right)$. By considering the Ansätze (77) and (78), we have $u^{(1)}_i=R_{ikl}\xi _{kl}+c_i$ and $p_s^{(0)}=S_{kl}\xi _{kl}+\phi p^{(0)}_f$, respectively. We also note that $\xi _{ij}=I^{S}_{ijkl}\xi _{kl}=\frac{1}{2}(\delta _{ik}\delta _{jl}+\delta _{il}\delta _{jk}) \xi _{kl}$, cf. Eq. (103).

The problem for the auxiliary third rank tensor $\mathcal {R}$ and second rank tensor ${\textsf{S}}$ (79a–79c) rewrites, component-wise, as follows

106a $$\begin{aligned}&\frac{\partial \left( 2\bar{\mu }_s\tilde{\xi }_{ijkl}\right) }{\partial y_j}-\frac{\partial (\delta _{ij}S_{kl})}{\partial y_j}=-\frac{\partial (2\bar{\mu }_s I^{S}_{ijkl})}{\partial y_j} \,\, \text{ in } \,\, \Omega _s, \end{aligned}$$

106b $$\begin{aligned}&\frac{\partial \mathcal {R}_{ikl}}{\partial y_i}=-\delta _{kl}\qquad \qquad \qquad \qquad \qquad \qquad \,\, \text{ in } \,\, \Omega _s, \end{aligned}$$

106c $$\begin{aligned}&\left( 2\bar{\mu }_s \tilde{\xi }_{ijkl}-\delta _{ij}S_{kl}\right) n_j=-2\bar{\mu }_sI^{S}_{ijkl}n_j \qquad \text{ on } \,\, \Gamma , \end{aligned}$$

where we have denoted by $\tilde{\xi }_{ijkl}$ the “*ijkl*” component of the fourth rank tensor $\tilde{\xi }_{{\textbf{y}}}(\mathcal {R})$, that is

107 $$\begin{aligned} \tilde{\xi }_{ijkl}=\frac{1}{2}\left( \frac{\partial R_{ikl}}{\partial y_j}+\frac{\partial R_{jkl}}{\partial y_i} \right) . \end{aligned}$$

## Footnotes

1 Relationship (87) is in some contexts expressed in terms of the fluid content, and variations of this latter are then equal to $-\nabla \cdot {\textbf{q}}$ by means of conservation of the fluid mass, as per equations (59) and (73) [24] or equivalently equation (11) and (12) as summarised in [51].
